# Exploring the landscape of clinical research in AATD: a cross-sectional review of registered studies in clinicaltrials.gov, clinicaltrialsregister.eu, and WHO ICTRP

**DOI:** 10.1186/s13023-026-04708-5

**Published:** 2026-11-12

**Authors:** Philipp Höger, Markus Ries, Arturo Olivares Rivera, Katharina Buschulte, Konstantina Kontogianni, Hilal Ersöz, Ralf Eberhardt, Sebastian Fähndrich, Sabina Janciauskiene, Felix Herth, Franziska C Trudzinski

**Affiliations:** 1 Department of Pneumology and Critical Care Medicine, Thoraxklinik University of Heidelberg, Röntgenstraße 1, 69126 Heidelberg, Germany; 2 Translational Lung Research Center (TLRC-H), German Center for Lung Research (DZL), Heidelberg, Germany; 3 Medical Faculty Heidelberg, Center for Pediatrics and Adolescent Medicine, Pediatric Neurology and Metabolic Medicine, Heidelberg University, in Neuenheimer Feld 430, 69120 Heidelberg, Germany; 4 Department of Pneumology and Critical Care Medicine, Asklepios Klinik Barmbek, Hamburg, Germany; 5 Department of Pulmonology, Medical Faculty, University Medical Center Freiburg, Freiburg, Germany; 6 Department of Respiratory Medicine, Hannover Medical School, Hannover, Germany; 7 Biomedical Research in Endstage and Obstructive Lung Disease Hannover (BREATH), Member of the German Center for Lung Research (DZL), Hannover, Germany

**Keywords:** Alpha1-antitrypsin deficiency, Research registry, Clinical trial

## Abstract

**Background:**

Since the approval of human alpha-1 proteinase inhibitor in 1987 for treating alpha-1 antitrypsin deficiency (AATD)-related emphysema, no new targeted therapies have been authorized for this condition. Notably, effective treatments addressing the extrapulmonary manifestations of AATD remain lacking. This review provides a comprehensive overview of the current landscape and progress in clinical development for AATD therapies.

**Methods:**

A comprehensive search of all registered trials was performed on 26 March 2025 across the web-based databases of ClinicalTrials.gov, Clinical Trials Register Europe, and the World Health Organization International Clinical Trials Registry Platform.

**Results:**

A total of 147 studies were identified. Of these, 112 (76.2%) were interventional studies and 35 (23.8%) were observational studies. The median actual enrollment in interventional studies was 33 participants, compared with a median planned enrollment of 55 participants. The pharmaceutical industry (57%) was the main source of financial support for these studies. The most prominent study locations were North America (51.7%) and Europe (42.9%), followed by Oceania (16.3%). Clinical research on AATD treatments includes human and recombinant protein therapies delivered intravenously, by inhalation or subcutaneously, as well as gene- and RNA-based therapies. Additional approaches, such as AAT protein folding correctors and neutrophil elastase inhibitors, are under investigation. Currently, RNA interference therapeutics are the most advanced therapeutic approach, with Phase 3 clinical trials ongoing.

**Conclusion:**

The development of novel therapies holds promise to broaden treatment options for patients with AATD, targeting both pulmonary and extrapulmonary manifestations.

**Clinical trial number:**

Not applicable

**Supplementary Information:**

The online version contains supplementary material available at https://doi.org/10.1186/s13023-026-04708-5.

## Introduction

Alpha-1 antitrypsin deficiency (AATD, OMIM #613490) is a rare autosomal-codominant inherited disorder primarily caused by mutations in the *SERPINA1 gene*, most commonly by the Z (c.1096G > A, p.E366K) and S (c.863 A > T, p.E288V) variants, which lead to reduced circulating levels and impaired function of alpha-1 antitrypsin (AAT) [1, 2]. The Z allele occurs at a frequency of approximately 2% in Northern European populations, compared with around 0.2% in African and East Asian populations [3]. Similarly, the S allele has a frequency of approximately 2% in most European populations, with notably higher frequencies of 10–12% in the Iberian Peninsula (Spain and Portugal) [4]. The most significant physiological function of AAT, an acute phase glycoprotein, is the protection of lung tissue from enzymatic degradation, with the inhibition of neutrophil proteases, specifically neutrophil elastase (NE) [1, 5] .In the context of AATD, an excess of proteases in the lungs can result in the development of emphysema in early adulthood, bronchiectasis and bronchial asthma [1]. However, it is important to recognize that extrapulmonary manifestations, particularly liver disease, can occur at any age in individuals with AATD [6, 7]. Beyond its anti-protease function, AAT also has immunomodulatory properties [1, 8, 9], which may contribute to increased susceptibility to infections such as pneumonia and bronchitis, as well as to other inflammatory conditions including panniculitis and vasculitis observed in patients with AATD.

In 1987, the U.S. Food and Drug Administration (FDA) approved human plasma-derived AAT to restore circulating AAT levels above the protective threshold and slow the progression of emphysema [10]. This therapy is administered once weekly at a standard dose of 60 mg per kilogram of body weight [10]. To date, intravenous augmentation therapy with plasma-derived AAT remains the only disease-specific treatment available for patients with AATD-related lung disease [10]. While its efficacy on extrapulmonary manifestations remains unclear, the evidence regarding its impact on pulmonary outcomes is also viewed as uncertain or debated in some countries, which in some cases has led to restricted reimbursement [11]. However, more recent registry data suggest a potential benefit in terms of prognosis, including improved survival [12–16]. Evidence for efficacy in panniculitis and systemic vasculitis is limited to case reports and small series, suggesting potential benefit but lacking controlled clinical trial validation [9, 17–19].

Several limitations are associated with approved augmentation therapy. It relies on human plasma donations, meaning its availability is directly tied to the population’s willingness to donate plasma regularly [20, 21]. Moreover, despite rigorous preparation procedures, there is still a risk of transmitting infection and the unintended introduction of additional plasma components remains [21]. Furthermore, the need for weekly visits to medical facilities can negatively impact patients’ quality of life (QoL) [22]. These challenges highlight the need to develop alternative therapeutic approaches that not only address pulmonary, but also extrapulmonary manifestations of AATD, with the goal of improving the overall QoL for affected patients.

To date, there have been review articles on the clinical development of orphan drugs for the treatment of AATD [23, 24]. However, a comprehensive analysis of the research status on AATD, encompassing both current and previously globally registered clinical trials, has not yet been conducted and represents a novel contribution. Alongside pharmacological studies for the treatment of pulmonary and extrapulmonary manifestations, non-pharmacological clinical studies were also considered. This review summarizes the status of therapeutic development for AATD-related diseases and discusses emerging opportunities to advance novel treatment strategies.

## Materials and methods

This work is a cross-sectional observational review. Its design, implementation, analysis, and reporting followed the STROBE (Strengthening the Reporting of Observational Studies in Epidemiology) guidelines [25], which sets a standard for reporting observational studies and promotes the completeness of the results through a checklist. Ethical approval was not required, as no individual patient data were collected or analyzed.

### Endpoints

The aim of this study was to determine the total number of registered interventional and observational studies in the field of AATD, providing a comprehensive overview of the scope and progress of clinical research in this area to date.

### Data sources

A comprehensive review of all registered trials was conducted on 26 March 2025 using three major web-based databases: the U.S. National Library of Medicine (clinicaltrials.gov), the European Union Clinical Trials Register (clinicaltrialsregister.eu) and the World Health Organization’s International Clinical Trials Registry Platform (WHO ICTRP). The latter is a meta-registry that currently comprises 18 different primary registries worldwide [26]. Although the European Union Clinical Trials Register is included within the ICTRP, it was analysed separately in the present study to ensure capture of potentially non-transferred records. The search terms “alpha-1-antitrypsin-deficiency” and “AATD” were used, without applying any filters.

For ClinicalTrials.gov, the search terms described above were entered into the “Condition/disease” search field. The fields “Other terms”, “Intervention/treatment”, and “Location” were left blank. Under “Study Status”, the option “All studies” was selected. No additional filters were applied.

For ClinicalTrialsRegister.eu, the search terms described above were entered into the designated search field. No additional filters were applied in the “Advanced Search” section.

In the ICTRP database, the search terms described above were likewise entered into the search field of the Search Portal. No filters were applied in the fields “Restrict to COVID-19”, “Search for clinical trials in children”, “Without Synonyms”, “Phases”, “With results only”, “Rare diseases/orphan drugs”, or “Genome editing”. No specific filters were applied in the “Advanced Search” section either.

### Inclusion criteria

All registered studies identified through the aforementioned search that either investigated the treatment of AATD as interventional trials or included exclusively patients with AATD as observational studies were included for the present analysis.

### Exclusion criteria

All registered studies identified in the above-described search that investigated indications other than AATD as interventional trials, or included patients without AATD as observational studies, were excluded from the present analysis. In addition, duplicate studies were excluded from the analysis. Studies were considered duplicates if all recorded parameters (Target, Agent, Phase, Enrollment, Age, Study Start, and Status) were identical. If studies differed in at least one of these parameters, they were considered separate studies and included in the analysis.

A total of 126 duplicate studies and 41 registered studies were excluded because they were either intervention studies with indications other than AATD, or observational studies that did not exclusively include patients with AATD. The interventional studies addressed indications other than AATD, for example: alcohol-associated hepatitis; Covid-19; acute-graft-versus-host disease; chronic pancreatitis; transplantation, lung rejection; type 1 diabetes mellitus; neuromyelitis optica; post cardiac surgery systemic inflammatory response; chronic fatigue syndrome; acute myocardial infarction; cystic fibrosis; HIV disease; liver disease. In the excluded observational studies, the cohorts were not limited to patients with AATD but additionally comprised individuals with COPD, other pulmonary diseases, liver diseases, women with pre-eclampsia, sarcoidosis, or ulcerative colitis, see supplementary Table 7.

### Definitions

The registered studies were categorized based on the biochemical properties, molecular mechanisms, or therapeutic approach of the drug, or the focus of the observational study, into the following groups: protein therapy, gene therapy, AAT protein folding corrector, NE inhibitor, other pharmacological and non-pharmacological therapies, diagnostic, database/prospective cohort studies, and “other”. The category “other” included studies that could not be classified into any of the aforementioned groups.

### Descriptive statistical analysis

Data extraction was performed, followed by a descriptive statistical analysis. The extracted variables included the type of agent, study design, phase of the study, registration number, actual/planned enrollment, age range of participants, study start date, current study status, location(s), and sponsor. Microsoft Office Professional Plus 2019 was used for the descriptive statistical analyses.

## Results

### Registered studies

A total of 147 different registered studies on the topic of AATD were identified, 112 (76.2%) of these were interventional studies and 35 (23.8%) observational studies. There were 149 hits on clinicaltrials.gov, 37 hits on clinicaltrialsregister.eu, and 128 hits on trialsearch.who.int.

### General findings

#### Sponsor

Of the 147 studies, 83 (56.5%) were sponsored by industry and 56 (38.1%) by academic institutions. Four studies (2.7%) were funded by private individuals, two (1.4%) by non-profit organisations, one (0.7%) by a lung forum, and one additional study (0.7%) had an unknown sponsor.

#### Planned enrollment

For the enrollment analysis, all studies with a reported enrollment greater than zero were included. Of the 112 interventional studies, this applied to 102 studies. A distinction was made between actual enrollment and planned enrollment, with only one of these two figures reported for each study. In 80 interventional studies, actual enrollment was reported, with a median of 33 participants (minimum: 3; maximum: 345, interquartile range: 75). In the remaining 22 interventional studies, planned enrollment was reported, with a median of 55 participants (minimum: 8; maximum: 290, interquartile range: 108).

Similarly, enrollment data were available for a total of 34 observational studies. Actual enrollment was reported for 17 studies, with a median of 100 participants (minimum: 6; maximum: 6655, interquartile range: 109). In a further 17 studies, planned enrollment was reported, with a median of 230 participants (minimum: 30; maximum: 50.000, interquartile range: 1923).

#### Locations

Out of 147 studies, 86 (58.5%) were located in a single country, 54 (36.7%) in multiple countries, and the location of 7 (4,8%) studies could not be detected. Of the 86 studies conducted in a single country, 42 were carried out in the United States of America, 11 in the United Kingdom, 7 in Germany, 6 in the Netherlands, 5 in Japan, 4 in France, 3 each in Spain and New Zealand, 2 in Ireland, and 1 each in Australia, Canada, and Czech Republic.

When multiple countries were involved, the United States of America was most frequently represented (33 studies), followed by EU countries and the United Kingdom (29 studies each). Australia (20 studies) and New Zealand (15 studies) were also commonly included, whereas the Russian Federation (5 studies) and South America (2 studies) appeared only in a small number of studies.

Geographically, North American countries were involved in 76 studies (51.7%), European countries in 63 studies (42.9%), Oceanian countries in 24 studies (16.3%), Asian countries in 5 studies (3.4%), South American in 2 (1.4%) and African countries in 0 studies (0.0%).

#### Study types

**Fig. 1:**
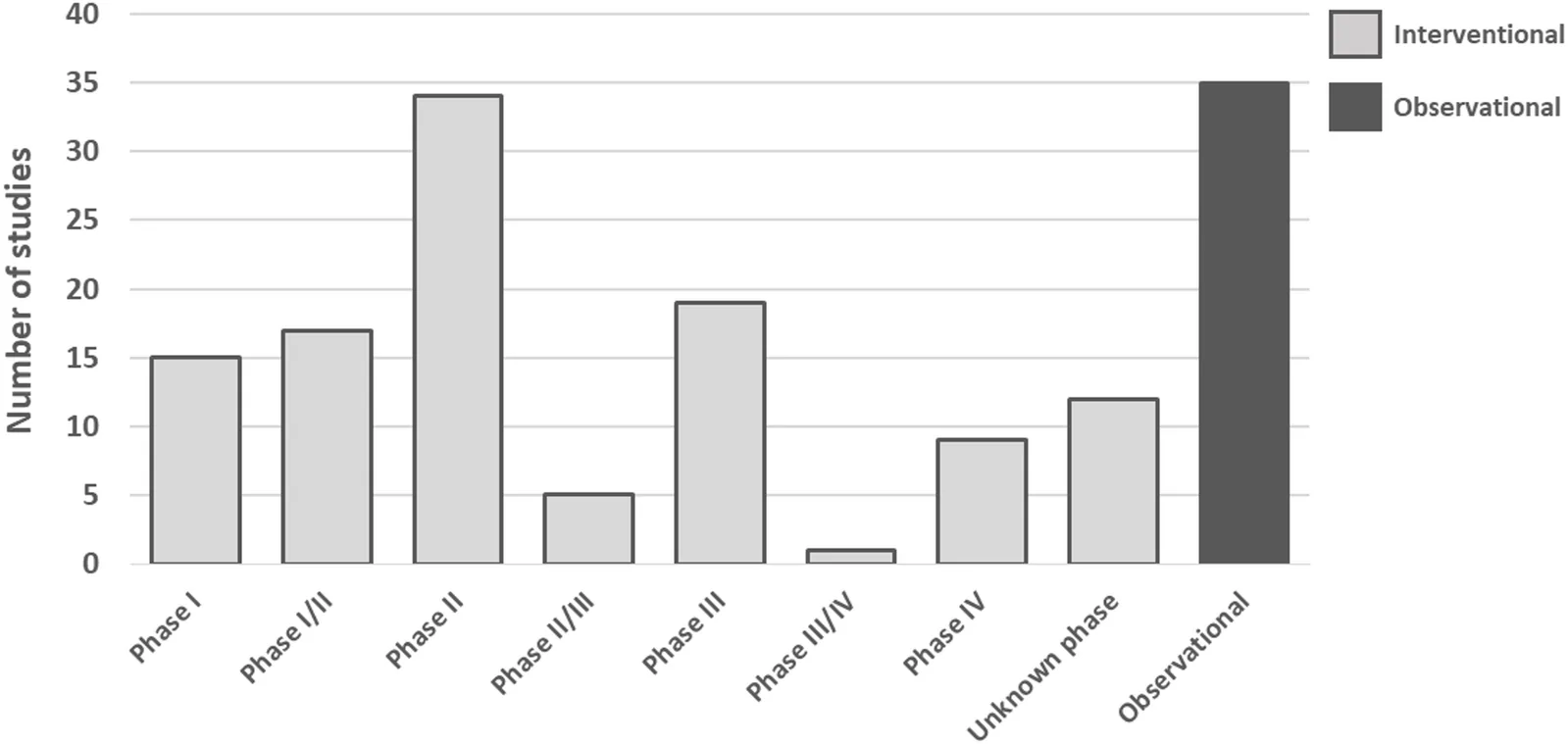
Distribution of studies by phase and study type

A clinical phase was specified in 100 of the 112 interventional studies, with the following distribution: 15 studies (13.4%) were in Phase I, 17 studies (15.2%) were in Phases I + II, 34 studies (30.4%) were in Phase II, 5 studies (4.5%) were in Phases II + III, 19 studies (17.0%) were in Phase III, one study (0.9%) was in Phases III/IV, nine studies (8.0%) were in Phase IV, and 12 studies (10.7%) had an unknown phase, see Fig. 1.

### Spectrum of clinical development

**Fig. 2:**
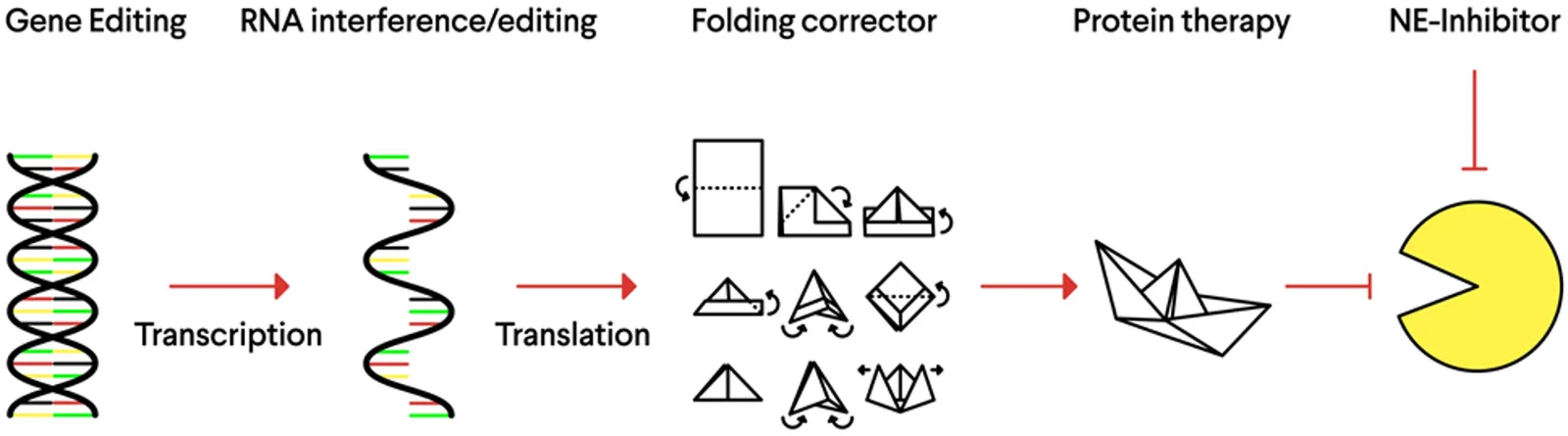
Simplified illustrative representation of therapeutic approaches currently in clinical development for the treatment of alpha-1 antitrypsin deficiency

The 147 identified studies were categorised into the following groups: protein therapy, gene therapy, AAT protein folding corrector, NE-inhibitor, other pharmacological and non-pharmacological therapies, diagnostic, database/prospective cohort studies, and other. The various therapeutic approaches currently in clinical development for the treatment of AATD are schematically illustrated in Fig. 2.

#### Protein therapy

A total of 51 out of 147 studies focused on protein therapy for the treatment of pulmonary manifestations of AATD. The clinical development landscape of protein therapies, as reflected in 51 studies, includes human alpha-1 proteinase inhibitor (A1-PI) therapy (27 studies), recombinant protein therapy (6 studies), investigations on optimal A1-PI dosing (6 studies), inhaled protein therapy (7 studies), protein therapy for the treatment of bronchiectasis in the context of AATD (2 studies), subcutaneous protein therapy (1 study), and home self-administration strategies (2 studies), see Table 1.

**Table 1:** Overview of registered studies on protein therapy

Target	Agent	Phase	Registration number	Enrollment	Age	Study start	Status
Protein therapy, human	Prolastin	3	EUCT 2024-515894-80-00	not mentioned	18 +	2025	recruitment pending
	Aralast	4	NCT05466747	0	18-75	2024	Withdrawn
	human AAT	not specified	JPRN-jRCT2080225207	3	20 +	2020	completed with results
	Prolastin C	1/2	NCT02870309	4	20 +	2016	Completed with results
	Prolastin C	1/2	NCT02870348	4	20 +	2016	Completed with results
	human AAT	1/2	JPRN-jRCT2080223139	3	20 +	2016	not recruiting, completed with results
	human AAT	1/2	JPRN-jRCT2080223105	3	20 +	2016	unknown
	GLASSIA	3	NCT02525861	34	18 +	2016	Completed with results
	Prolastin C	3	EudraCT 2015-004110-23	250	18 +	2016	not recruiting
	Prolastin C	3	NCT02796937/EudraCT 2024-513962-20-00	290	20-72/18 +	2016	Enrolling by invitation
	Prolastin C	2/3	NCT02282527	32	18-70	2014	Completed with results
	GLASSIA	4	NCT01651351	30	18-65	2012	Completed with results
	Alfalastin	x	NCT01676688*	17	18 +	2012	Completed
	Zemaira	4	NCT00670007	140	18 +	2008	Completed with results
	Prolastin	2	EudraCT 2007-004869-18	not applicable	18 +	2008	Completed with results
	Zemaira	3	EudraCT 2007-007129-38	140	18 +	2008	Completed with results
	x	4	EudraCT 2008-001962-94	8	18 +	2008	ongoing
	Kamada-AAT	2/3	NCT00460096	50	18 +	2007	Completed
	Zemaira	4	NCT00261833	180	18-65	2006	Completed with results
	Prolastin C	3	NCT00301366	38	18 +	2006	Completed with results
	Prolastin C	3	NCT00295061	24	18 +	2006	Completed with results
	Prolastin C	3	EudraCT 2005-002402-36	35	18 +	2006	Completed
	Zemaira	3/4	EudraCT 2005-003459-12	180	18 +	2006	Completed with results
	Aralast	4	NCT00313144	127	18 +	2006	Terminated with results
	ARALAST	4	NCT00396006	21	18 +	2006	Completed with results
	ARALAST	1	NCT00242385	25	18 +	2005	Completed with results
	Prolastin	2	NCT00263887	77	18 +	2003	Completed with results
Protein therapy, inhaled	Kamada AAT	3	EUCTR 2019-000602-30-GB	220	18-64	2020	Trial now transitioned, no results available
	Kamada AAT	3	OMON55372	70	18-64	2019	Recruiting
	Kamada-AAT	3	NCT04204252EUCT 2024-516054-21-00	220	18-65	2019	Recruiting
	Kamada-AAT	2	NCT02001688	36	18-65	2014	Completed with results
	Kamada AAT	2/3	NCT01217671	168	18 +	2009	Completed
	Kamada AAT	2/3	EudraCT 2008-005326-36	200	18 +	2009	Completed with results
	Aerosolized, Recombinant AAT	1/2	NCT00161707	40	18 +	2003	Completed
Protein therapy, inhaled (Bronchiectasis)	Prolastin-C Liquid	4	NCT05582798	20	18 +	2022	Completed
	Prolastin	not specified	NCT03383939	20	18-65	1996	Completed
Protein therapy, subcutaneous	Human AAT 15% subcutaneous	1/2	NCT04722887	16	18-80	2021	Active, not recruiting
Protein therapy, recombinant	INBRX-101	2	NCT05897424/EudraCT 2023-508137-14-00	130	18-80 18-84	2024	Recruiting
	INBRX-101	2	NCT05856331/EudraCT 2023-508084-76-00	99	18-80/18-84	2023	Active, not recruiting
	OsrhAAT	1	NCT05315921	48	18-55	2022	Unknown status
	INBRX-101	1	NCT03815396	31	18-80	2019	Completed
	OctaAlpha1	2	NCT03385395	0	18 +	2018	Withdrawn
	aerosolized, recombinant AAT	1/2	NCT00157092	15	18 +	2004	Completed
Self-therapy at home	Respreeza	X	NCT04262284*	60	18 +	2019	Active, not recruiting
	Alpha-1 augmentation therapy in the home through Coram Specialty Infusion	X	NCT01974830*	120	18 +	2013	Completed
Single dose vs. double dose	Double dose of Aralast	4	NCT04440488	0	18-65	2021	Withdrawn
	Double dose of Aralast NP and Glassia	3	NCT02722304	7	18 +	2016	Terminated with results
	Double Dose of human AAT	3	EudraCT 2013-001870-38	339	18 +	2014	completed
	Double Dose of human AAT	3	NCT01983241/EudraCT 2023-510030-83-00	345	18-70/18 +	2013	Active, not recruiting
	Double Dose of human AAT	2	NCT01669421	10	18-75	2012	Completed with results
	Double dose of Prolastin C	2	NCT01213043	30	18-70	2010	Completed with results

*Observational study

#### Gene therapy

Out of 147 studies, a total of 37 (25.2%) focused on gene therapy for the treatment of AATD. The clinical development landscape of gene therapies comprises gene editing (4 studies), vector-based therapy (7 studies), RNA editing (3 studies), and RNA interference therapeutics (23 studies), see Table 2.

**Table 2:** Overview of registered studies on gene therapy/AAT protein folding corrector

Target	Agent	Phase	Registration number	Enrollment	Age	Study start	Status
Adeno-associated virus vector therapy	ADVM-043	x	NCT03804021*	6	18 +	2018	Completed
	ADVM-043	1/2	NCT02168686	6	18 +	2017	Completed with results
	rAAV1-CB-hAAT	2	NCT01054339	9	18-75	2010	Completed with results
	rAAV1-CB-hAAT	2	EudraCT 2009-014286-20	9	18 +	2010	Completed with results
	rAAV1-CB-hAAT	1	NCT00430768	9	18 +	2006	Completed with results
HSV-1-associated vector therapy	Inhaled KB408 (Nebulization)	1	NCT06049082	12	18-70	2024	Recruiting
Gene editing	NTLA-3001 (CRISPR/Cas9)	1/2	EudraCT 2023-508138-33-00	0	18 +	2024	Withdrawn
	NTLA-3001 (CRISPR/Cas9)	1/2	NCT06622668	0	18-75	2024	Withdrawn
Gene editing, base-editing therapy to correct the PiZ mutation	BEAM-302	1/2	NCT06389877	106	18-70	2024	Recruiting
	BEAM-302	1/2	EudraCT 2023-509256-34-00	106	18-64	2024	Recruiting
Gene transfer	normal AAT genes into muscle cells	1	NCT00377416	12	18 +	2004	Completed
RNA editing oligonucleotide	KRRO-110	1/2	NCT06677307	64	18 -70	2025	Recruiting
RNA editing oligonucleotide (A-to-I)	WVE-006	1/2	NCT06405633/EudraCT 2024-511981-36-00	24	18-70/18 +	2024	Recruiting
	WVE-006	1	NCT06186492	56	18-65	2023	Recruiting
RNA interference	Fazirsiran	3	NCT06165341	50	18 -75	2024	Recruiting
	Fazirsiran	3	CTIS2023-504198-19-00	49	18 +	2024	Recruiting
	Fazirsiran	3	EudraCT 2023-503497-21-00	40	18-84	2023	Recruiting
	Fazirsiran	3	NCT05899673	37	18 +	2023	Active, not recruiting
	Fazirsiran	3	NCT05677971/EudraCT 2022-501943-34-00	160	18-75/18-84	2023	Recruiting
	Belcesiran	2	EudraCT 2021-005217-14	54	18 +	2022	Prematurely Ended
	Belcesiran	2	EudraCT 2020-003313-35	46	18 +	2022	Completed with results
	Belcesiran	2	NL-OMON55922	3	18-99	2022	not recruiting
	Belcesiran	2	NCT05146882	0	18-75	2021	Withdrawn
	Belcesiran	2	NCT04764448	16	18-75	2021	Terminated with results
	Fazirsiran	2/3	EudraCT 2018-003385-14	36	18 +	2019	Completed with results
	Fazirsiran	2	NCT03946449	16	18-75	2019	Completed
	Fazirsiran	2	NCT03945292	40	18-75	2019	Completed with results
	Fazirsiran	2	EudraCT 2019-000068-86	16	18 +	2019	Completed with results
	Belcesiran	1	NCT04174118	30	18-55	2019	Completed
	ALN-AAT02	1/2	NCT03767829	32	18-65	2018	Terminated
	ALN-AAT	1/2	EudraCT 2018-001362-41	96	18-64	2018	Completed with results
	Fazirsiran	1	NCT03362242	45	18-55	2018	Completed
	ARC-AAT	2	NCT02900183	0	18-75	2016	Withdrawn
	ARC-AAT	2	EudraCT 2016-000917-59	12	18 +	2016	Prematurely Ended
	ALN-AAT	1/2	EudraCT 2015-001297-18	66	18 +	2015	Completed with results
	ALN-AAT	1	NCT02503683	26	18-65	2015	Terminated
	ARC-AAT Injection	1	NCT02363946	65	18-70	2015	Terminated with results
AAT protein folding corrector	BMN 349	1	NCT06738017	12	18 - 64	2024	Recruiting
	VX-864	2	NCT05643495	14	18-80	2023	Completed
	VX-864	2	EudraCT 2022-002746-40-IE	20	18 and older	2023	Prematurely Ended, no results available
	VX-668	1	NCT05727800	114	18-55	2023	Completed
	VX-634	1	NCT05579431	127	18-55	2022	Completed
	VX-864	2	NCT04474197	44	18-80	2020	Completed with results
	VX-814	2	NCT04167345/EudraCT 2019-003650-92	48	18-80	2020	Terminated with results
	VX-864	2	EUCTR 2019-004881-16-IE	40	18 and older	2020	Completed with results
	ZF874	1	NCT04443192	69	18-72	2020	Terminated
	4-phenyl butyric acid	2	NCT00067756	12	18-65	2001	Completed

*Observational study

#### AAT protein folding corrector

A total of 10 out of 147 registered studies in the early phase of clinical development address the use of AAT correctors. The majority of the studies were recorded for the AAT corrector VX-864, **see** Table 2.

#### NE-Inhibitor and alternative therapeutic approaches

Among 147 clinical studies, four focused on the development of neutrophil elastase (NE) inhibitors as potential treatments for AATD. In addition to NE inhibitors, various alternative therapeutic strategies are being explored to address different aspects of AATD. One study investigated a selective retinoic acid receptor gamma (RARγ) agonist aimed at promoting alveologenesis and lung tissue repair. Mucolytic agents were evaluated to improve mucus clearance in affected airways. Therapies targeting pulmonary microvascular blood flow, including arginine succinate supplementation (ASS), were explored to enhance lung perfusion. Strategies to reduce portal venous flow were assessed for potential benefits in managing liver-related complications. Vitamin K administration was studied for its role in reducing elastin degradation and preserving lung elasticity. Interventional approaches included two studies on endoscopic lung volume reduction: one using valves and another using coils to improve lung function in emphysematous patients with AATD. Furthermore, three clinical trials examined the effectiveness of pulmonary rehabilitation programs tailored specifically for patients with AATD, focusing on improving exercise capacity and QoL, **see** Table 3.

**Table 3:** Overview of registered studies on neutrophil elastase inhibitor and other pharmacological or non-pharmacological therapies

Target	Agent	Phase	Registration number	Enrollment	Age	Study start	Status
Neutrophil Elastase Inhibitor	PHP-303	2	EudraCT 2019-002501-22	60	18 and older	2020	Unknown status
Neutrophil elastase inhibitor, oral	Alvelestat	2	NCT03679598	63	18-80	2019	Completed with results
	Alvelestat	2	NCT03636347	99	18-75	2018	Completed
	Alvelestat	2	EudraCT 2018-001309-95	182	18 and older	2018	Completed with results
Alveologenesis	RO3300074 (Retinoic Acid Receptor gamma Agonist)	2	EudraCT 2004-001688-23	300	18 and older	2004	Completed with results
Bronchoscopy	Lung Volume Reduction Coil (PneumrX Inc.)	not specified	NCT02273349	0	18-99	2014	Withdrawn
	Endoscopic Lung Volume Reduction	not specified	NCT01357460	18	30 and older	2011	Terminated
Endothelial function	ASS	2	NCT03008915	15	40 and older	2017	Completed with results
Mucolytic	Hyaluronic Acid Inhalation Solution	2	NCT03114020	27	18-80	2017	Completed
Rehabilitation	Exercise training	not specified	NCT03802357	30	40-80	2019	Unknown status
	Pulmonary rehabilitation	not specified	NCT02915614	40	25-75	2017	Unknown status
	Effects of Exercise Training	not specified	NCT01380626	20	40-75	2010	Completed
Treatment to reduce portal pressure	Carbamazepine	2	NCT01379469	20	14-80	2012	Terminated with results
Vitamin	Vitamin K	x	NCT03285100*	30	18 and older	2017	Unknown status

*Observational study

#### Diagnostic, “Others”, Database/Prospective cohort studies

7 studies focused on the diagnostics of AATD, exploring areas such as genetic sequencing, imaging, microbiome, and the assessment of emphysema severity, see supplementary Table 4.

In the category “Other”, 10 studies focus on various aspects of lung research in the context of AATD. These include reasons for genetic testing, comorbidities, environmental factors, epidemiology, epigenetics and immunology, genomic research, immune response in lung diseases, the pathobiology of early lung destruction, and prognostic biomarkers, see supplementary Table 5.

In addition, a total of 18 registered observational studies were identified. Among these, seven patient registries were established, including a comprehensive DNA and tissue database. Six observational studies aimed to identify prognostic biomarkers for lung or liver disease progression in the context of AATD. Other studies focused on genetic factors predisposing individuals to liver injury, the consequences of delayed diagnosis, the clinical outcomes of patients receiving AAT augmentation therapy, the natural history of AATD-related liver disease, and the prevalence of AATD in different populations, see supplementary Table 6.

### Clinical phases

**Fig. 3:**
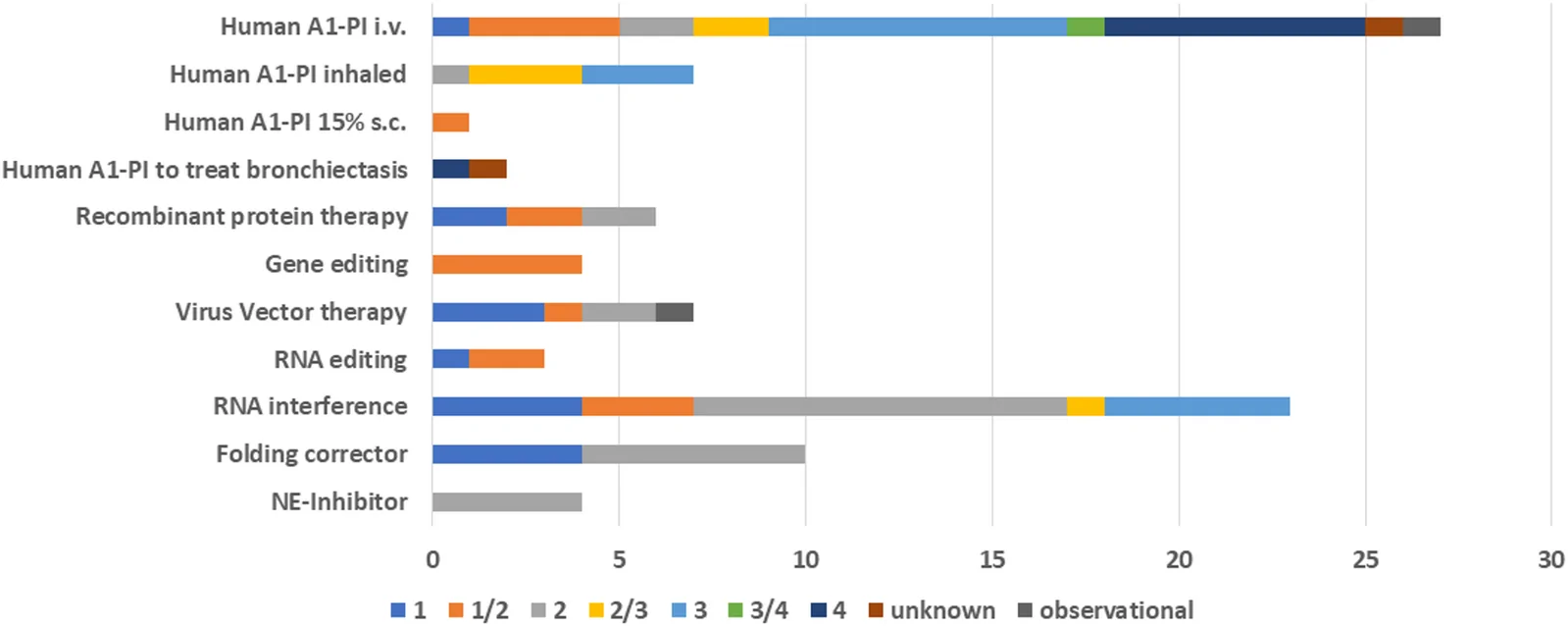
Illustration of the clinical phases of the key compounds currently in clinical development for the treatment of AATD

The following Fig. 3 illustrates the clinical phases of the key compounds that are currently in clinical development for the treatment of AATD.

## Discussion

This study provides an integrative overview of the current clinical research landscape in AATD, highlighting not only the spectrum of therapeutic interventions under investigation but also key trends in study design, funding, and geographic distribution. By contextualizing these findings, our analysis offers a critical perspective on how present research directions may inform and shape future treatment strategies for AATD. Clinical research in AATD has primarily focused on pharmacological approaches, with protein therapy (34%) and gene therapy (25%) representing the predominant areas of investigation.

The majority of the studies were intervention trials (76.2%). Planned enrollment numbers in these intervention studies were relatively small, which is an expected limitation given the rarity of AATD. The implications of small sample sizes for statistical power depend on factors such as the study design, the primary endpoint, and the expected effect size. However, collaborative, multicenter, and potentially international efforts may help generate more robust evidence for future therapeutic strategies.

The predominance of studies conducted in industrialized countries such as the United States of America is not unexpected, given that these settings typically benefit from greater financial resources and more highly developed research infrastructures. In addition, stable institutions in industrialized nations provide clear legal and ethical frameworks, which are essential for conducting studies. Furthermore, many headquarters of companies researching AATD are located in the United States of America, which likely also explains why most studies are conducted there.

The sole approved therapy for AATD remains intravenous alpha-1 proteinase inhibitor augmentation [10]. Ongoing research is also investigating alternative modes of administration, including subcutaneous application (Phase 1/2) and inhaled formulations (Phase 3) of human alpha-1 proteinase inhibitors. As an example of an inhaled AAT therapy, Kamada AAT can be mentioned, which was in Phase 3 clinical development at the time of data collection. Following completion of the data acquisition for the present study, the pharmaceutical company developing this inhaled AAT announced in December 2025 that the trial had been prematurely terminated due to failure to meet its primary endpoint. FEV1 was used as the primary outcome measure to assess emphysema progression in the study. However, several studies have shown that the emphysema index, derived from quantitative computed tomography, is a more sensitive marker for assessing emphysema progression [14, 27]. Consequently, in studies specifically investigating emphysema progression, quantitative CT parameters, such as the emphysema index or PD15, may be more sensitive endpoints for assessing structural disease progression than FEV₁.

In some countries, such as the United States of America, home self-therapy is carried out predominantly by outpatient care services and only rarely by the patients themselves. Enhanced patient education would be important to help reduce the burden on outpatient care resources. By offering less invasive and more convenient routes of therapy administration, these approaches may enhance treatment adherence and patient satisfaction compared with traditional intravenous therapy. Inhaled therapy would allow patients to self-administer treatment at home, eliminating the need for weekly visits to medical facilities. Subcutaneous administration offers an additional option for patients with difficult venous access. However, inhaled protein therapy faces the challenge of maintaining protein stability, as exposure to air can result in denaturation and loss of biological activity [28]. Another potential limitation of subcutaneous administration is the relatively high volume of fluid required [29]. Recombinant protein therapies offer the advantage of a lower risk of pathogen transmission by eliminating the need for human plasma-derived products. Furthermore, use of recombinant AAT allows for structural modifications that may enhance biochemical activity and improve therapeutic efficacy. An example of this is INBRX-101, a recombinant human AAT-Fc fusion protein, which combines AAT with the Fc region of immunoglobulin G, prolonging circulation time and enhancing serum concentrations [30]. These innovative formulations highlight the potential for more convenient, safer, and effective AATD therapies, ultimately improving patient adherence and clinical outcomes.

Registered studies are also focusing on determining the optimal dosage for augmentation therapy. The RAPID study showed that annual lung density loss is lower with higher serum AAT levels [14]. Furthermore, a double dose of 120 mg/kg body weight once a week has already been shown to be well tolerated [31]. The placebo-controlled, randomised SPARTA study is currently underway and aims to compare the efficacy of the standard and double doses [32].

In recent years, gene therapy has gained increasing significance in the clinical development of treatments for AATD. At the level of DNA, therapeutic approaches are currently being explored to edit the *SERPINA1* gene to restore the most common mutation associated with severe AATD. Furthermore, vector-based therapies have a role in clinical research. Initial research concentrated on adeno-associated vectors. These are distinguished by their low pathogenicity and a wide range of serotypes, which enable transduction into various target organs [33]. However, this method is limited by the restricted genomic capacity of approximately 4.6 kb [33]. The present study investigates the potential of HSV-associated vectors, which offer a significantly higher genomic capacity of about 152 kb, thus opening up new therapeutic possibilities [33]. Vector-based therapies do not cure the disease itself but act as delivery systems to transport therapeutic agents (DNA/RNA) into target cells. In the case of the inhaled therapy KB408, an HSV-based vector is used to deliver a functional human *SERPINA1 gene* into the nuclei of lung epithelial cells.

At the RNA-level, both RNA-editing agents and RNA interference (RNAi) therapeutics are under investigation. RNA editing agents aim to correct disease-causing mutations at the mRNA level through the selective substitution of specific bases, thereby restoring the original, functional RNA sequence [34]. In the context of AATD, this approach enables the production of correctly folded AAT protein.

Mutant AAT undergoes misfolding and polymerisation within hepatocytes, leading to the formation of intracellular polymers that induce hepatotoxic stress and may result in liver diseases such as cirrhosis or hepatocellular carcinoma [1]. RNA interference therapeutics promote the cleavage or degradation of specific target mRNA molecules, thereby preventing their translation and ultimately inhibiting the production of mutant, misfolded proteins [35]. By reducing the synthesis of mutant AAT, RNA interference therapeutics decrease polymer formation and thereby alleviate hepatotoxic stress [36]. However, it should be noted that a potential disadvantage of this RNA interference-based therapeutic approach is that it may lead to a reduction in serum AAT levels, since mutant AAT retains some residual functional activity [37]. Consequently, this could result in an elevated risk of developing pulmonary emphysema associated with AATD.

RNAi-based therapies already reached Phase 3 trials, reflecting their advanced stage of clinical development. The predominance of registered studies on RNAi therapeutics highlights this approach as a major focus of current research [36]. In contrast, folding stabilizers and AAT protein folding correctors are still in the early stages of clinical development. Their aim is to prevent the misfolding of mutated AAT proteins.

While DNA-level strategies aim to correct permanently the underlying *SERPINA1* mutation, RNA-level interventions act transiently to inhibit the synthesis of mutated and misfolded proteins without altering the genome. This distinction means that RNA-targeted therapies can rapidly reduce pathogenic protein levels and are generally transient [38], whereas DNA-targeted approaches have the potential for long-lasting correction but face greater challenges related to delivery, durability, and safety in clinical settings [39].

Another class of pharmacological treatment of AATD are NE inhibitors. By directly targeting NE, these therapies aim to reduce the proteolytic damage to lung tissue that underlies emphysema development in patients with AATD. In particular, oral administration of NE inhibitors could offer a convenient option for patients with poor venous access or those who value treatment autonomy, potentially improving adherence and overall QoL. Moreover, NE inhibitors could complement existing augmentation therapies by providing an additional mechanism to preserve lung function and slow disease progression.

In addition to therapeutic options, several patient registries, such as the EARCO (European Alpha-1 Research Collaboration) registry, have been established to collect and track clinical and radiomorphological data from patients with AATD over time. These registries are of significant importance, as one of the remaining open questions in AATD is why patients with the same genotype — particularly those homozygous for the Z mutation, the most common variant associated with severe AATD — exhibit such variable clinical courses, especially with regard to emphysema progression. Moreover, these data sources play a crucial role in evaluating the long-term effects of augmentation therapy. Furthermore, several observational studies have been conducted to identify prognostic biomarkers for the progression of pulmonary or hepatic manifestations of AATD.

### Limitations

A limitation of this study is that it was restricted to three trial registries: the U.S. National Library of Medicine (clinicaltrials.gov), the European Union Clinical Trials Register (clinicaltrialsregister.eu), and the WHO International Clinical Trials Registry Platform (WHO ICTRP), which include 18 national and regional registers. Relevant studies listed only in alternative or less commonly used registries may therefore have been missed, potentially leading to an underestimation of study diversity. In addition, for 10.7% of trials, the clinical phase was not specified, limiting the ability to accurately assess their stage of development. Future studies could address these limitations by expanding the search to additional regional or specialty registries and by encouraging greater standardization and completeness of trial reporting.

Due to the search strategy and the biomedical focus of the study, only a limited number of qualitative studies were included. Studies examining the psychological burden that may arise from a chronic disease with potential multi-organ manifestations and the associated loss of work capacity would be helpful. Considering that augmentation therapy is not available in some countries due to the absence of reimbursement policies, studies analyzing the societal and financial consequences of denying patients with AATD access to this therapy would also be of importance.

This work provides an overview of the clinical development landscape of therapeutic agents for the treatment of AATD as of 26 March 2025. In recent years, significant advances have been made in the field of AATD. Consequently, by the time of publication of this manuscript, new study results or additional therapeutics currently in clinical development may already be available.

## Conclusion

In summary, beyond the approved human protein augmentation therapy, a broad spectrum of novel treatments for AATD is under clinical investigation. These include DNA- and RNA-based strategies, recombinant protein formulations with enhanced biochemical activity, and small-molecule neutrophil elastase inhibitors. Alternative routes of administration, such as oral and inhaled delivery, are also being developed to improve patient convenience and adherence. RNA interference therapeutics represent one of the most advanced investigational liver-directed modality, with Phase 3 clinical trials ongoing. Looking ahead, combining RNA interference with established augmentation therapy may represent a potential strategy to address both pulmonary and extrapulmonary manifestations of AATD. However, the potential benefits of such combination approaches remain hypothetical and require further investigation.

## Supplementary Information

Below is the link to the electronic supplementary material.

Supplementary Material 1

## Data Availability

The datasets utilised in the study are available from the corresponding author upon reasonable request.

## Abbreviations

AAT: Alpha 1-Antitrypsin
AATD: Alpha 1-Antitrypsin deficiency
A1-PI: Alpha-1-Proteinase-inhibitor
CT: Computed tomography
NE: Neutrophil elastase
QoL: Quality of life
RNAi: RNA interference
WHO ICTRP: World health organization's international clinical trials registry platform

## References

[1] Strnad P, McElvaney NG, Lomas DA. Alpha1-Antitrypsin Deficiency. N Engl J Med. 2020;382:15, 1443–55. 10.1056/NEJMra1910234 32268028

[2] Foil KE. Variants of SERPINA1 and the increasing complexity of testing for alpha-1 antitrypsin deficiency. Therapeutic Adv chronic disease. 2021;12:33–48. 10.1177/20406223211015954 PMC8367212 34408833

[3] Blanco I, Bueno P, Diego I, Pérez-Holanda S, Casas-Maldonado F, Esquinas C, Miravitlles M. Alpha-1 antitrypsin Pi*Z gene frequency and Pi*ZZ genotype numbers worldwide: an update. Int J Chronic Obstr Pulm Dis. 2017;12:561–9. 10.2147/COPD.S125389 PMC5315200 28243076

[4] Blanco I, de Serres FJ, Fernandez-Bustillo E, Lara B, Miravitlles M. Estimated numbers and prevalence of PI*S and PI*Z alleles of alpha1-antitrypsin deficiency in European countries. Eur Respir J. 2006;27(1):77–84. 10.1183/09031936.06.00062305 16387939

[5] Dasí F. Alpha-1 antitrypsin deficiency. Med Clin. 2024;162:7, 336–42. 10.1016/j.medcli.2023.10.014 37993348

[6] Hagiwara M, Divino V, Munnangi S, Delegge M, Park S, Marins EG, Ren K, Strange C. Retrospective Database Analysis of Liver-Related Clinical Events in Adult and Pediatric Patients with Alpha-1 Antitrypsin Deficiency in the United States. Hepatic medicine: Evid Res. 2024;16:55–64. 10.2147/HMER.S469769 PMC11283783 39070302

[7] Craig TJ, Corbett ML, Meadows JA. Improving Detection of Alpha-1 Antitrypsin Deficiency: Role of the Allergist. J allergy Clin Immunol Pract. 2023;11:8, 2348–54. 10.1016/j.jaip.2023.04.027 37146883

[8] Campos MA, Geraghty P, Holt G, Mendes E, Newby PR, Ma S, Luna-Diaz LV, Turino GM, Stockley RA. The Biological Effects of Double-Dose Alpha-1 Antitrypsin Augmentation Therapy. A Pilot Clinical Trial. Am J Respir Crit Care Med. 2019;200:3, 318–26. 10.1164/rccm.201901-0010OC PMC6680306 30965011

[9] Mazzuca C, Vitiello L, Travaglini S, Maurizi F, Finamore P, Santangelo S, Rigon A, Vadacca M, Angeletti S, Scarlata S. Immunological and homeostatic pathways of alpha – 1 antitrypsin: a new therapeutic potential. Front Immunol. 2024;15:1443297. 10.3389/fimmu.2024.1443297 PMC11366583 39224588

[10] Wewers MD, Casolaro MA, Sellers SE, Swayze SC, McPhaul KM, Wittes JT, Crystal RG. Replacement therapy for alpha 1-antitrypsin deficiency associated with emphysema. N Engl J Med. 1987;316:17, 1055–62. 10.1056/NEJM198704233161704 3494198

[11] Brantly M. Treatment for Alpha-1 Antitrypsin Deficiency: Does Augmentation Therapy Work? Am J Respir Crit Care Med. 2023;208:9, 948–9. 10.1164/rccm.202309-1585ED PMC10870859 37724887

[12] Seersholm N, Wencker M, Banik N, Viskum K, Dirksen A, Kok-Jensen A, Konietzko N. Does alpha1-antitrypsin augmentation therapy slow the annual decline in FEV1 in patients with severe hereditary alpha1-antitrypsin deficiency? Wissenschaftliche Arbeitsgemeinschaft zur Therapie von Lungenerkrankungen (WATL) alpha1-AT study group. Eur Respir J. 1997;10:10, 2260–3. 10.1183/09031936.97.10102260 9387950

[13] The Alpha-1-Antitrypsin Deficiency Registry Study Group. Survival and FEV1 decline in individuals with severe deficiency of alpha1-antitrypsin. The Alpha-1-Antitrypsin Deficiency Registry Study Group. Am J Respir Crit Care Med. 1998;158(1):49–59. 10.1164/ajrccm.158.1.9712017 9655706

[14] Chapman KR, Burdon JGW, Piitulainen E, Sandhaus RA, Seersholm N, Stocks JM, Stoel BC, Huang L, Yao Z, Edelman JM, McElvaney NG. Intravenous augmentation treatment and lung density in severe α1 antitrypsin deficiency (RAPID): a randomised, double-blind, placebo-controlled trial. Lancet (London England). 2015;386:9991, 360–8. 10.1016/S0140-6736(15)60860-1 26026936

[15] McElvaney NG, Burdon J, Holmes M, Glanville A, Wark PAB, Thompson PJ, Hernandez P, Chlumsky J, Teschler H, Ficker JH, Seersholm N, Altraja A, Mäkitaro R, Chorostowska-Wynimko J, Sanak M, Stoicescu PI, Piitulainen E, Vit O, Wencker M, Tortorici MA, Fries M, Edelman JM, Chapman KR. Long-term efficacy and safety of α1 proteinase inhibitor treatment for emphysema caused by severe α1 antitrypsin deficiency: an open-label extension trial (RAPID-OLE). Lancet Respiratory Med. 2017;5:1, 51–60. 10.1016/S2213-2600(16)30430-1 27916480

[16] Fraughen DD, Ghosh AJ, Hobbs BD, Funk G-C, Meischl T, Clarenbach CF, et al. Augmentation therapy for severe alpha-1 antitrypsin deficiency improves survival and is decoupled from spirometric decline—a multinational registry analysis. Am J Respir Crit Care Med. 2023;208(9):964–974. 10.1164/rccm.202305-0863OC PMC10870866 37624745

[17] Blanco I, Lara B, de Serres F. Efficacy of alpha1-antitrypsin augmentation therapy in conditions other than pulmonary emphysema. Orphanet J Rare Dis. 2011;6:14. 10.1186/1750-1172-6-14 PMC3094201 21486454

[18] Stockley RA. The multiple facets of alpha-1-antitrypsin. Annals translational Med. 2015;3:10, 130. 10.3978/j.issn.2305-5839.2015.04.25 PMC4486914 26207223

[19] Baranovski BM, Schuster R, Nisim O, Brami I, Lior Y, Lewis EC. Alpha-1 Antitrypsin Substitution for Extrapulmonary Conditions in Alpha-1 Antitrypsin Deficient Patients. Chronic Obstr pulmonary Dis (Miami Fla. 2018;5(4):267–76. 10.15326/jcopdf.5.4.2017.0161 PMC6361480 30723784

[20] Petrache I, Hajjar J, Campos M. Safety and efficacy of alpha-1-antitrypsin augmentation therapy in the treatment of patients with alpha-1-antitrypsin deficiency. Biologics: targets therapy. 2009;3:193–204. 10.2147/btt.2009.3088 PMC2726081 19707408

[21] Cai K, Gierman TM, Hotta J, Stenland CJ, Lee DC, Pifat DY, Petteway SR. Ensuring the biologic safety of plasma-derived therapeutic proteins: detection, inactivation, and removal of pathogens. BioDrugs: Clin immunotherapeutics biopharmaceuticals gene therapy. 2005;19:2, 79–96. 10.2165/00063030-200519020-00002 PMC7099715 15807628

[22] Sandhaus RA, Strange C, Zanichelli A, Skålvoll K, Koczulla AR, Stockley RA. Improving the Lives of Patients with Alpha-1 Antitrypsin Deficiency. Int J Chronic Obstr Pulm Dis. 2020;15:3313–22. 10.2147/COPD.S276773 PMC7735792 33328731

[23] Trudzinski FC, Presotto MA, Buck E, Herth FJF, Ries M. Orphan drug development in alpha-1 antitypsin deficiency. Sci Rep. 2022;12:1. 10.1038/s41598-022-19707-2 PMC9477815 36109566

[24] Höger P, Ries M, Olivares Rivera A, Ersöz H, Buschulte K, Fähndrich S, Kontogianni K, Herth F, Trudzinski FC. Advances in orphan drug development for alpha-1 antitrypsin deficiency: a 2025 update from the FDA and EMA. Ther Adv Respir Dis. 2026;20:17534666251411227. 10.1177/17534666251411227 PMC12779913 41499187

[25] Vandenbroucke JP, von Elm E, Altman DG, Gøtzsche PC, Mulrow CD, Pocock SJ, Poole C, Schlesselman JJ, Egger M. Strengthening the Reporting of Observational Studies in Epidemiology (STROBE): explanation and elaboration. Epidemiol (Cambridge Mass). 2007;18(6):805–35. 10.1097/EDE.0b013e3181577511 18049195

[26] World Health Organization. ICTRP Registry Network [Internet]. World Health Organization; [cited 2025 Mar 26]. https://www.who.int/ictrp/network/en/.

[27] Green CE, Parr DG, Edgar RG, Stockley RA, Turner AM. Lung density associates with survival in alpha 1 antitrypsin deficient patients. Respir Med. 2016;112:81–7. 10.1016/j.rmed.2016.01.007 26874894

[28] Matthews AA, Ee PLR, Ge R. Developing inhaled protein therapeutics for lung diseases. Mol Biomed. 2020;1:1. 10.1186/s43556-020-00014-z PMC7595758 34765995

[29] Bittner B, Richter W, Schmidt J. Subcutaneous Administration of Biotherapeutics: An Overview of Current Challenges and Opportunities. BioDrugs: Clin immunotherapeutics biopharmaceuticals gene therapy. 2018;32:5, 425–40. 10.1007/s40259-018-0295-0 PMC6182494 30043229

[30] Brantly ML, Kuhn BT, Farah HW, Mahadeva R, Cole A, Chang CL, Brown CD, Campos MA, Lascano JE, Babcock EK, Bhagwat SP, Boyea TF, Veldstra CA, Andrianov V, Kalabus JL, Eckelman BP, Veale AG. Recombinant Alpha-1 Antitrypsin-Fc Fusion Protein INBRX-101 in Adults With Alpha-1 Antitrypsin Deficiency: A Phase 1 Study. Chronic Obstr pulmonary Dis (Miami Fla). 2024;11(3):282–92. 10.15326/jcopdf.2023.0469 PMC11216232 38809792

[31] Campos MA, Kueppers F, Stocks JM, Strange C, Chen J, Griffin R, Wang-Smith L, Brantly ML. Safety and pharmacokinetics of 120 mg/kg versus 60 mg/kg weekly intravenous infusions of alpha-1 proteinase inhibitor in alpha-1 antitrypsin deficiency: a multicenter, randomized, double-blind, crossover study (SPARK). COPD. 2013;10(6):687–695. 10.3109/15412555.2013.800852 23862647

[32] Sorrells S, Camprubi S, Griffin R, Chen J, Ayguasanosa J. SPARTA clinical trial design: exploring the efficacy and safety of two dose regimens of alpha1-proteinase inhibitor augmentation therapy in alpha1-antitrypsin deficiency. Respir Med. 2015;109(4):490–9. 10.1016/j.rmed.2015.01.022 25727857

[33] McLachlan G, Alton EWFW, Boyd AC, Clarke NK, Davies JC, Gill DR, Griesenbach U, Hickmott JW, Hyde SC, Miah KM, Molina CJ. Progress in Respiratory Gene Therapy. Hum Gene Ther. 2022;33:17–8. 10.1089/hum.2022.172 PMC7615302 36074947

[34] Erion DM, Liu LY, Brown CR, Rennard S, Farah H. Editing Approaches to Treat Alpha-1 Antitrypsin Deficiency. Chest. 2025;167(2):444–52. 10.1016/j.chest.2024.09.038 39401571

[35] Alshaer W, Zureigat H, Karaki A, Al-Kadash A, Gharaibeh A, Hatmal L, Aljabali MM, A. A. A., and, Awidi A. siRNA: Mechanism of action, challenges, and therapeutic approaches. Eur J Pharmacol. 2021;905:174178. 10.1016/j.ejphar.2021.174178 34044011

[36] Clark VC, Strange C, Strnad P, Sanchez AJ, Kwo P, Pereira VM, van Hoek B, Barjaktarevic I, Corsico AG, Pons M, Goldklang M, Gray M, Kuhn B, Vargas HE, Vierling JM, Vuppalanchi R, Brantly M, Kappe N, Chang T, Schluep T, Zhou R, Hamilton J, Martin S, J., and, Loomba R. Fazirsiran for Adults With Alpha-1 Antitrypsin Deficiency Liver Disease: A Phase 2 Placebo Controlled Trial (SEQUOIA). Gastroenterology. 2024;167:5, 1008–e10185. 10.1053/j.gastro.2024.06.028 38964420

[37] Llewellyn-Jones CG, Lomas DA, Carrell RW, Stockley RA. The effect of the Z mutation on the ability of alpha 1-antitrypsin to prevent neutrophil mediated tissue damage. Biochim Biophys Acta. 1994;1227:3, 155–60. 10.1016/0925-4439(94)90089-2 7986822

[38] Booth BJ, Nourreddine S, Katrekar D, Savva Y, Bose D, Long TJ, Huss DJ, Mali P. RNA editing: Expanding the potential of RNA therapeutics. Mol therapy: J Am Soc Gene Therapy. 2023;31(6):1533–49. 10.1016/j.ymthe.2023.01.005 PMC9824937 36620962

[39] Lummerstorfer M, Lächelt U. Non-Viral CRISPR carriers: transient delivery with lasting effects. Drug Delivery. 2026;33:1. 10.1080/10717544.2026.2614125 PMC12818331 41543272

