# Supplementary material for: Exploring the landscape of clinical research in AATD: a cross-sectional review of registered studies in clinicaltrials.gov, clinicaltrialsregister.eu, and WHO ICTRP
Source: Orphanet J Rare Dis. 2026 Nov 12;21:417. doi: 10.1186/s13023-026-04708-5 (PMC13761029; doi:10.1186/s13023-026-04708-5)
Supplement: Supplementary file 1 [file 13023_2026_4708_MOESM1_ESM.docx]

**Supplement**

| **Target** | **Agent** | **Phase** | **Registration number** | **Enrollment** | **Age** | **Study start** | **Status** |
| --- | --- | --- | --- | --- | --- | --- | --- |
| **Genetic Sequencing** | **Cohort** | **x** | [**NCT02810327**](https://clinicaltrials.gov/study/NCT02810327?cond=NCT02810327&viewType=Card&rank=1)***** | **117** | **18 and older** | **2015** | **Completed with results** |
| **Imaging** | **Lung MRI scan** | **x** | [**NCT04966221**](https://clinicaltrials.gov/study/NCT04966221?cond=NCT04966221&viewType=Card&rank=1)***** | **75** | **18 and older** | **2022** | **Unknown status** |
|  | **Magnetic resonance imaging (MRI) using inhaled hyperpolarized 129Xe gas** | **not specified** | [**NCT03455686**](https://clinicaltrials.gov/study/NCT03455686?cond=NCT03455686&viewType=Card&rank=1) | **60** | **18-85** | **2018** | **Recruiting** |
|  | **Positron emission tomography (PET) scanning** | **not specified** | [**ISRCTN80875207**](https://trialsearch.who.int/Trial2.aspx?TrialID=ISRCTN80875207) | **30** | **unknown** | **2008** | **not recruiting** |
|  | **High resolution computed tomography (CT) scans** | **x** | [**NCT00532805**](https://clinicaltrials.gov/study/NCT00532805?cond=NCT00532805&viewType=Card&rank=1)***** | **49** | **18 and older** | **2007** | **Completed** |
| **Microbioma** | **Microbioma in sputa** | **x** | [**NCT02547532**](https://clinicaltrials.gov/study/NCT02547532?cond=NCT02547532&viewType=Card&rank=1)***** | **100** | **18 and older** | **2015** | **Completed** |
| **Determine the severity of emphysema** | **CX3CR1 receptors** | **x** | [**NL-OMON49069**](https://trialsearch.who.int/Trial2.aspx?TrialID=NL-OMON49069)***** | **56** | **18-99** | **2020** | **not recruiting** |

**Supplementary Table 4:** Overview of registered diagnostic studies. *Observational study.

| **Target** | **Agent** | **Phase** | **Registration number** | **Enrollment** | **Age** | **Study start** | **Status** |
| --- | --- | --- | --- | --- | --- | --- | --- |
| **Alpha-1 Coded Testing** | **Evaluation of the population demographics, reasons for testing, and outcomes** | **x** | [**NCT00500123**](https://clinicaltrials.gov/study/NCT00500123?cond=NCT00500123&viewType=Card&rank=1)***** | **50000** | **all ages** | **2001** | **Recruiting** |
| **Comorbidities** | **Prevalence of diverticular disease in patients with AATD** | **x** | [**NL-OMON47360**](https://trialsearch.who.int/Trial2.aspx?TrialID=NL-OMON47360)***** | **230** | **18-99** | **2017** | **Recruiting** |
| **Comorbidities** | **Prevalence of diverticular disease in patients with AATD** | **x** | [**NL-OMON28920**](https://onderzoekmetmensen.nl/en/trial/28920)***** | **230** | **not mentioned** | **2017** | **recruiting** |
| **Environmental factors** | **Environmental impact on physical performance** | **not applicable** | [**NCT02502201**](https://clinicaltrials.gov/study/NCT02502201?cond=NCT02502201&viewType=Card&rank=1) | **27** | **18 and older** | **2015** | **Terminated** |
| **Epidemiology** | **Prevalence** | **not applicable** | [**NCT02382367**](https://clinicaltrials.gov/study/NCT02382367?cond=NCT02382367&viewType=Card&rank=1) | **190** | **18-80** | **2014** | **Completed** |
| **Epigenetics and immunology** | **Epigenetic regulation of immunity** | **x** | [**NCT02691611**](https://clinicaltrials.gov/study/NCT02691611?cond=NCT02691611&viewType=Card&rank=1)***** | **39** | **18-85** | **2015** | **Unknown status** |
| **Genomic Research** | **Examine the interaction between the microflora in the lower airway and the concentration of AAT** | **x** | [**NCT01832220**](https://clinicaltrials.gov/study/NCT01832220?cond=NCT01832220&viewType=Card&rank=1)***** | **140** | **40-80** | **2013** | **Completed** |
| **Immune response in lung diseases** | **Evaluation of macrophage function** | **x** | [**NCT01851642**](https://clinicaltrials.gov/study/NCT01851642?cond=NCT01851642&viewType=Card&rank=1)***** | **220** | **18 and older** | **2007** | **Recruiting** |
| **Pathobiology** | **Pathobiology of early lung destruction** | **x** | [**NCT00001462**](https://clinicaltrials.gov/study/NCT00001462?cond=NCT00001462&viewType=Card&rank=1)***** | **60** | **all ages** | **1995** | **Completed** |
| **Prognostic biomarkers** | **Identify clinical and biological prognostic factors** | **not applicable** | [**NCT01862211**](https://clinicaltrials.gov/study/NCT01862211?cond=NCT01862211&viewType=Card&rank=1) | **296** | **7 and older** | **2013** | **Completed** |

**Supplementary Table 5:** Overview of registered studies that do not belong to any other category. *Observational study.

| **Target** | **Agent** | **Phase** | **Registration number** | **Enrollment** | **Age** | **Study start** | **Status** |
| --- | --- | --- | --- | --- | --- | --- | --- |
| **Database** | **EARCO-Registry** | **x** | [**NCT04180319**](https://clinicaltrials.gov/study/NCT04180319?cond=NCT04180319&viewType=Card&rank=1)***** | **3000** | **all ages** | **2020** | **Unknown status** |
| **Database** | **Alpha-1 Research Registry** | **x** | [**NCT04157049**](https://clinicaltrials.gov/study/NCT04157049?cond=NCT04157049&viewType=Card&rank=1)***** | **4000** | **all ages** | **2019** | **Recruiting** |
| **Database** | **Czech AATD Registry** | **x** | [**NCT05178277**](https://clinicaltrials.gov/study/NCT05178277?cond=NCT05178277&viewType=Card&rank=1)***** | **300** | **all ages** | **2018** | **Recruiting** |
| **Database** | **Patient Registry for liver disease in AATD** | **x** | [**NCT02929940**](https://clinicaltrials.gov/study/NCT02929940?cond=NCT02929940&viewType=Card&rank=1)***** | **500** | **18-99** | **2015** | **Unknown status** |
| **Database** | **Alpha-1 Foundation DNA and Tissue Bank** | **x** | [**NCT00884455**](https://clinicaltrials.gov/study/NCT00884455?cond=NCT00884455&viewType=Card&rank=1)***** | **5000** | **all ages** | **2002** | **Enrolling by invitation** |
| **Database** | **Alpha-1 Foundation Research Registry** | **x** | [**NCT00499941**](https://clinicaltrials.gov/study/NCT00499941?cond=NCT00499941&viewType=Card&rank=1)***** | **6655** | **all ages** | **2000** | **Completed** |
| **Database** | **Alpha1-antitrypsin Deficiency Registry** | **x** | [**NCT00005292**](https://clinicaltrials.gov/study/NCT00005292?cond=NCT00005292&viewType=Card&rank=1)***** | **x** | **0-100** | **1988** | **Completed** |
| **Genetic traits** | **genetic traits that predispose to liver injury** | **x** | [**NCT00005098**](https://clinicaltrials.gov/study/NCT00005098?cond=NCT00005098&viewType=Card&rank=1)***** | **160** | **0 and older** | **1993** | **Terminated** |
| **Impact of delayed diagnosis** | **cohort** | **x** | [**NCT03039335**](https://clinicaltrials.gov/study/NCT03039335?cond=NCT03039335&viewType=Card&rank=1)**i*** | **43** | **18 and older** | **2016** | **Completed** |
| **Monitoring** | **Clinical course of patients that received AAT infusion** | **x** | [**NCT04799873**](https://clinicaltrials.gov/study/NCT04799873?cond=NCT04799873&viewType=Card&rank=1)***** | **10** | **18-110** | **2020** | **Completed** |
| **Natural History of AATD-liver disease** | **cohort** | **x** | [**NCT06512454**](https://clinicaltrials.gov/study/NCT06512454?cond=NCT06512454&viewType=Card&rank=1)***** | **1000** | **18 and older** | **2024** | **Recruiting** |
| **Prevalence** | **cohort** | **x** | [**NCT01419158**](https://clinicaltrials.gov/study/NCT01419158?cond=NCT01419158&viewType=Card&rank=1)***** | **3457** | **18 and older** | **2008** | **Completed** |
| **Prognostic biomarkers** | **PiMZ-cohort** | **x** | [**NCT06505603**](https://clinicaltrials.gov/study/NCT06505603?cond=NCT06505603&viewType=Card&rank=1)***** | **80** | **18 and older** | **2025** | **Not yet recruiting** |
| **Prognostic biomarkers** | **PiZZ cohort** | **x** | [**NCT05297812**](https://clinicaltrials.gov/study/NCT05297812?cond=NCT05297812&viewType=Card&rank=1)***** | **286** | **18 and older** | **2022** | **Active, not recruiting** |
| **Prognostic biomarkers (Initiation of Augmentation Therapy)** | **cohort** | **x** | [**DRKS00013943**](https://trialsearch.who.int/Trial2.aspx?TrialID=DRKS00013943)***** | **50** | **all ages** | **2018** | **not recruiting** |
| **Prognostic biomarkers (liver disease)** | **cohort** | **x** | [**NCT02014415**](https://clinicaltrials.gov/study/NCT02014415?cond=NCT02014415&viewType=Card&rank=1)***** | **120** | **18 and older** | **2013** | **Active, not recruiting** |
| **Prognostic biomarkers (liver disease)** | **PiZZ-Cohort** | **x** | [**NCT01810458**](https://clinicaltrials.gov/study/NCT01810458?cond=NCT01810458&viewType=Card&rank=1)***** | **109** | **18-70** | **2013** | **Completed** |
| **Prognostic biomarkers (emphysema)** | **cohort** | **x** | [**NCT00700934**](https://clinicaltrials.gov/study/NCT00700934?cond=NCT00700934&viewType=Card&rank=1)***** | **300** | **18 and older** | **2005** | **Unknown status** |

**Supplementary Table 6:** Overview of registered patient registries and prospective cohort studies. *Observational study.

| **Target** | **Agent** | **Phase** | **Registration number** | **Enrollment** | **Age** | **Study start** | **Status** |
| --- | --- | --- | --- | --- | --- | --- | --- |
| **Protein therapy, human** | **Human A1-PI**  **Condition: Alcohol-Associated Hepatitis** | **2** | **NCT06582329** | **16** | **18 and older** | **2025** | **Not yet recruiting** |
|  | **Aralast**  **Condition: Covid-19** | **3** | **NCT04675086** | **0** | **18 and older** | **2021** | **Withdrawn** |
|  | **Prolastin-C Liquid Condition: Covid-19** | **2** | **NCT04547140** | **57** | **18 and older** | **2021** | **Terminated** |
|  | **Human A1-PI**  **Condition: Acute-graft-versus-host Disease** | **2/3** | **NCT03805789** | **310** | **12 and older** | **2019** | **Recruiting** |
|  | **Human A1-PI**  **Condition: Graft vs Host Disease** | **2** | **NCT01304537** | **30** | **18 and older** | **2018** | **Completed** |
|  | **Prolastin-C,**  **Condition: chronic pancreatitis** | **1/2** | **NCT02947087** | **48** | **18 and older** | **2017** | **Completed** |
|  | **Glassia**  **Condition: Acute-graft-versus-host Disease** | **not specified** | **NCT03172455** | **Not specified** | **All ages** | **2017** | **No longer available** |
|  | **Glassia**  **Condition: Transplantation, Lung Rejection** | **2** | **NCT02614872** | **30** | **18 and older** | **2016** | **Completed** |
|  | **Aralast NP** | **1/2** | **NCT02520076** | **5** | **18-68** | **2015** | **Completed** |
|  | **Human A1-PI**  **Condition: type 1 diabetes mellitus** | **2** | **NCT02093221** | **76** | **6-35** | **2014** | **Terminated with results** |
|  | **Human A1-PI**  **Condition: Neuromyelitis Optica** | **1** | **NCT02087813** | **0** | **18-75** | **2014** | **Withdrawn** |
|  | **Human A1-PI**  **Condition: Post Cardiac Surgery Systemic Inflammatory Response** | **1/2** | **NCT02191839** | **20** | **40-70** | **2014** | **Unknown status** |
|  | **Glassia**  **Condition: type 1 diabetes mellitus** | **2** | **NCT02005848** | **70** | **8-25** | **2014** | **Completed** |
|  | **Human A1-PI**  **Condition: Chronic fatigue syndrome** | **2** | **EudraCT 2014-002007-13** | **26** | **18 and older** | **2014** | **Prematurely Ended** |
|  | **Zemaira**  **Condition: Graft vs Host Disease** | **2** | **NCT01700036** | **40** | **18 and older** | **2013** | **Completed** |
|  | **Human A1-PI**  **Condition: Acute Myocardial Infarction** | **1/2** | **NCT01936896** | **10** | **21-99** | **2013** | **Completed with results** |
|  | **Glassia**  **Condition: type 1 diabetes mellitus** | **1/2** | **NCT01304537** | **24** | **10-25** | **2011** | **Completed** |
|  | **Human A1-PI, liquid**  **Condition: cystic fibrosis** | **1** | **NCT01347190** | **25** | **18-65** | **2011** | **Completed** |
|  | **Aralast NP**  **Condition: type 1 diabetes mellitus** | **1** | **NCT01319331** | **12** | **6-45** | **2010** | **Completed** |
|  | **Aralast NP**  **Condition: type 1 diabetes mellitus** | **2** | **NCT01183455** | **0** | **8-35** | **2010** | **Withdrawn** |
|  | **Aralast NP**  **Condition: type 1 diabetes mellitus** | **1** | **NCT01183468** | **17** | **8-35** | **2010** | **Terminated** |
|  | **Zemaira**  **Condition: HIV disease** | **2/3** | **NCT01370018** | **4** | **30-70** | **2006** | **Completed** |
| **Protein therapy, human, inhaled** | **Human A1-PI, inhaled**  **Condition: Cystic fibrosis** | **2** | **NCT01684410** | **30** | **18 and older** | **2012** | **Completed** |
|  | **Human A1-PI, inhaled**  **Condition: Bronchiolitis Obliterable Syndrome** | **2** | **NCT01394835** | **40** | **18-70** | **2011** | **Unknown status** |
|  | **Human A1-PI, inhaled**  **Condition: Cystic fibrosis** | **2** | **NCT00499837** | **21** | **5 and older** | **2007** | **Completed** |
|  | **Human A1-PI, inhaled**  **Condition: Dystic fibrosis** | **2** | **NCT02010411** | **17** | **14 and older** | **2004** | **Terminated** |
| **Prognostic biomarker** | **COPD cohort** | **x** | **NCT03823443*** | **7** | **35-80** | **2018** | **Terminated** |
| **Biomarker** | **Cohort of candidates for allogeneic transplantation for the first time** | **x** | **NCT03188601*** | **27** | **18 and older** | **2018** | **Completed** |
| **Natural history and progression of cholestatic liver diseases** | **Cohort with Alagille syndrome, AATD, PFIC or BRIC and bile acid synthesis defects** | **x** | **NCT00571272*** | **1675** | **0-25** | **2007** | **Suspended** |
| **Liver Stiffness Measurement** | **Cohort with biliary Atresia, Alagille Syndrome, AATD, portal hypertension, liver fibrosis, cholestasis** | **x** | **NCT02922751*** | **552** | **0-21** | **2016** | **Completed** |
| **Alpha1 Antitrypsin level** | **Cohort of Women with Pre-eclampsia** | **x** | **NCT05622838*** | **160** | **All ages** | **2022** | **Unknown status** |
| **Genomic Research** | **Sarcoidosis cohort** | **x** | **NCT01831739*** | **368** | **18-85** | **2013** | **Completed** |
| **Transplant Suitability** | **Cohort of pulmonary diseases** | **not applicable** | **NCT01615484** | **24** | **15 and older** | **2013** | **Completed** |
| **Transplant Suitability** | **Cohort of pulmonary diseases** | **2** | **NCT01241942** | **11** | **18 and older** | **2010** | **Terminated** |
| **Non-Surgical Periodontal Therapy** | **Periodontitis Patients with or without Acute Coronary Syndrome** | **x** | **NCT04785235*** | **43** | **35-55** | **2007** | **Completed** |
| **Prevalence of lung function impairment** | **Cohort of Gambian women aged 15 and older** | **x** | **NCT06029543*** | **350** | **15 and older** | **2023** | **Not yet recruiting** |
| **Alterations in the contact activation system** | **Cohort of Ulcerative colitis** | **x** | **NCT04621006*** | **102** | **18 and older** | **2021** | **Completed** |
| **Antioxidants** | **Cohort of patients with lymphomas, breast cancer, ovarian cancer, and brain gliomas** | **x** | **NCT03470857*** | **83** | **18 and older** | **2017** | **Completed** |
| **Newborn screening** | **Cohort of newborn between birth and 28 days of life** | **x** | **NCT05687474*** | **6000** | **Up to 28 days** | **2022** | **Recruiting** |
| **Pharmacokinetic of mycophenolate mofetil** | **Mycophenolate Mofetil,**  **lung allograft recipients** | **3** | **EudraCT 2009-012231-15** | **70** | **18 and older** | **2009** | **Completed** |
| **G-CSF** | **G-CSF**  **Condition: Liver Disease** | **2** | **EudraCT 2009-010335-41** | **81** | **18 and older** | **2009** | **Completed** |

**Supplementary Table 7:** Overview of excluded registered patient registries and prospective cohort studies. *Observational study.
